# Functional Characterization of Suppressor of Cytokine Signalling 6 and Its Interaction with Erythropoietin Receptor in Colorectal Cancer Cells

**DOI:** 10.3390/cancers18010171

**Published:** 2026-01-04

**Authors:** Asma Al-Bahri, Fahad Zadjali, Shika Hanif, Zaina Alharthi, Hussein Sakr, Amira Al-Kharusi

**Affiliations:** 1 College of Medicine and Health Sciences, Sultan Qaboos University, P.O. Box 35, Muscat PC 123, Oman; abahri@squ.edu.om (A.A.-B.); s.hanif@squ.edu.om (S.H.); s143514@student.squ.edu.om (Z.A.); hsakr@squ.edu.om (H.S.); 2 Dean Office, Oman College of Health Sciences, P.O. Box 393, Muscat PC 113, Oman; fahad.alzadjali@ochs.edu.om

**Keywords:** suppressor of cytokine signalling (SOCS), suppressor of cytokine signalling 6 (SOCS6), erythropoietin receptor (EPOR), colorectal cancer (CRC), viability, migration, invasion, apoptosis, caspase-3

## Abstract

**Background:** Suppressor of Cytokine Signalling 6 (SOCS6) is a cytokine signalling suppressor that regulates receptor tyrosine kinase pathways by promoting degradation of signalling proteins, thereby controlling cell growth and survival. One of these tyrosine kinase receptors, Erythropoietin Receptor (EPOR), plays a critical role in CRC progression by enhancing tumour metabolism, angiogenesis, proliferation, and growth. This study investigates the molecular mechanisms governing SOCS6’s role in CRC pathogenesis using in vitro cell models and examines its interaction with EPOR expression following gene knockdown. **Methods:** Bioinformatics interaction between SOCS6 and EPOR were investigated using molecular visualization. HT-29 and COLO 320DM colorectal cancer cells were transfected with SOCS6 siRNA followed by measurement of SOCS6 and EPOR expression levels by qRT-PCR. The selected knockdown concentration was used in functional assays assessing cell viability, colony formation, migration, apoptosis, and invasion. **Results:** Bioinformatic results showed interaction between SOCS6 and EPOR through polar bonds. Furthermore, SOCS6 silencing increased cell viability and colony formation in both cell lines and significantly enhanced migration in COLO 320DM cells. Active caspase-3 levels were elevated markedly in HT-29 cells post SOCS6 knockdown, consistent with caspase-3’s reported oncogenic role in CRC. Moreover, EPOR knockdown selectively altered SOCS6 expression in HT-29 cells, indicating a regulatory feedback loop. EPOR silencing elevated cell viability at 24 h in both cell lines but caused a significant decrease in COLO 320DM cells at 72 h. **Conclusions:** These findings identify the SOCS6–EPOR axis as a potential target for personalized CRC therapy, supporting SOCS6’s tumour-suppressive and diagnostic roles.

## Simple Summary

Suppressor of Cytokine Signalling 6 (SOCS6) is a cytokine signalling suppressor that regulates receptor tyrosine kinase pathways to control cell growth and survival and has been implicated as a tumour suppressor in colorectal cancer. Erythropoietin Receptor (EPOR) plays a significant role in promoting tumour proliferation and angiogenesis in colorectal cancer (CRC). However, their interaction remains unexamined, and the roles of SOCS6 and EPOR in CRC are poorly understood. This study investigates the molecular mechanisms of SOCS6 in CRC pathogenesis and its association with EPOR expression after gene knockdown. The findings reveal that reducing SOCS6 increases cancer cell survival and migration, while changes in EPOR expression influence SOCS6 levels and cell survival differently depending on the cell type. This research highlights the complex relationship between SOCS6 and EPOR in colorectal cancer and suggests that targeting their interaction may offer new, personalized treatment strategies in colorectal cancer.

## 1. Introduction

Suppressor of cytokine signalling (SOCS) protein family consists of eight members: SOCS1 to SOCS7 and CIS, each with a unique SH2 domain, variable amino-terminal domain, and carboxyl-terminal SOCS box. These proteins have a conserved 40-amino-acid sequence in their C-terminal sections and varying N-terminal regions. Each SOCS domain has specific roles, such as binding to phosphotyrosine residues, suppressing kinase activity, and recruiting ubiquitin ligase machinery [1,2,3,4,5,6]. SOCS proteins regulate cellular signalling pathways, acting as negative feedback inhibitors, suppressing specific cytokine signals and restoring cellular homeostasis by blocking JAK activity or targeting the receptor complex for proteasomal degradation [2].

SOCS6 inhibits tumour growth in a variety of cancers, including glioblastoma, bladder carcinoma, non-small cell lung, prostate, lung, oesophageal squamous cell carcinoma, cervical, and gastric cancer [7,8,9,10,11,12,13]. The mechanism is mediated through inhibition of angiogenesis, decreasing cell proliferation, and increasing apoptosis [12]. When SOCS6 expression is downregulated or deleted in experimental designs, it impacts on cancer progression via epigenetic pathways like changes in DNA methylation [14,15]. Lower SOCS6 levels are associated with poor prognosis in several cancer types [10,16,17,18,19,20,21,22,23]. There are various small non-coding RNAs (miRNAs, lncRNAs, and circRNAs) that regulate SOCS6 expression; this, in turn, affects cancer progression and the efficacy of therapeutic interventions [14,24,25,26,27,28,29,30,31,32]. Additionally, SOCS6 regulates cancer cell susceptibility to radiation and chemotherapy, potentially suppressing tumour growth in various cancers through epigenetic and non-coding RNA processes [9,12,15,29,33]. Previous studies showed that SOCS6 can bind to specific Phosphorylated tyrosine residues in certain receptor tyrosine kinases, like FLT3 and c-KIT. This binding process lead to the downregulation of signalling cascades that normally promote cell growth and survival [34,35]. This phosphotyrosine-dependent binding is mediated by the SH2 domain of SOCS6. It usually recognizes certain amino acids patterns around the phosphorylated tyrosine residues, with a valine residue at the pY+1 position and hydrophobic residue at the pY+2 and pY+3 location being its main preferences [3]. Interestingly, there are possible SH2 domain binding sites within the intracellular tail of the erythropoietin receptors (EPOR), a key cytokine receptor that play in erythropoiesis and is being more known for its involvement in the survival and proliferation of cancer cells. Sequence analysis shown that the cytoplasmic domain of EPOR has many conserved tyrosine residues that are located within consensus pattern for SH2 domain binding [36]. These residues may act as SOCS6 docking sites indicating a possible interaction for the negative regulation mediated by SOCS6 for the EPOR signalling in colorectal cancer cells.

The erythropoietin receptor (EPOR) is a cytokine receptor with three distinct structural domains that plays a crucial role in cancer development. It is expressed in various non-hematopoietic tissues, including myoblasts, the central nervous system, endothelial cells, cardio-myocytes, solid tumours, uterus, retina, and liver [37,38,39,40]. EPOR’s role extends beyond erythropoiesis, it also plays a role in carcinogenesis [41,42,43,44]. EPOR’s presence in cancer cells highlights the complex network of signalling channels controlling cellular activity. It is found to play a significant role in the tumourigenic environment in various cancer types, including renal, colorectal, breast, and lung carcinomas [45,46,47]. When active, EPOR triggers processes within cells that contribute to tumour development, facilitating cell survival, proliferation, and angiogenesis [48,49]. EPOR’s dual function allows it to promote or protect against cancerous transformation depending on the circumstances [46,47]. Its potential therapeutic effects have been a topic of concern, with erythropoiesis-stimulating agents (ESAs) targeting EPOR to improve anaemia in cancer patients after chemotherapy [50]. However, concerns have been raised about the potential promotion of tumour growth and metastasis by activating signalling pathways mediated by EPOR. Thus, EPOR plays a pivotal role in cancer development and progression, with its intricate relationship highlighting its importance in tumourigenic environments.

As per the World Health Organization (WHO), Colorectal cancer (CRC) is the third most frequently diagnosed cancer worldwide and the second leading cause of cancer-related deaths [51]. The majority of CRCs are sporadic, influenced by both hereditary and environmental factors [52]. The primary pathways leading to CRC include the suppressor pathway, mutator pathway, and aberrant hyper methylation [53]. Lifestyle, environment, and heredity all play a role in the development of colorectal cancer. Diagnostic markers like carcinoembryonic antigen (CEA) and molecular markers like microsatellite instability (MSI) are crucial for diagnosis, prognosis, and treatment [54,55]. Mutations in genes like APC, KRAS, and TP53, as well as dysregulation of pathways like Wnt, Ras-MAPK, and PI3K-Akt, contribute to the disease’s progression. These molecular complexities highlight the role of genetic and signalling abnormalities in colorectal cancer [53,56]. SOCS6 expression is downregulated in CRC tissues and loss of SOCS6 contributes to enhanced oncogenic signalling, increased cell proliferation, and reduced apoptosis, promoting tumour growth and progression [16,57]. However, the detailed mechanistic studies in CRC are limited in the literature. Understanding these pathways has led to novel treatment targets, diagnostic markers, and cancer response indicators.

This study aims to understand the molecular mechanisms that regulate the involvement of SOCS6 in the pathogenesis of colorectal cancer through in vitro cell models, and to investigate its regulatory interaction with EPOR expression following gene knockdown.

## 2. Materials and Methods

### 2.1. Bioinformatics

Several software tools were utilized in a comprehensive bioinformatics strategy to study the interaction connections between SOCS6 and EPOR. We started by getting the 3D models of SOCS6 and EPOR from UniProt (Release 2025_04), which had the protein databases for both proteins. In order to get the protein structures ready for docking, PyMOL (Version 3.0.3) was used for protein preparation. The proteins were then subjected to energy minimization using Chimera (Version 1.17.3) or SWISS-PDB Viewer (Version 4.1) to guarantee that the structures were in their most energy-efficient conformations. Then, in order to find possible interaction areas on the surface of the proteins, binding site prediction was performed using SPPIDER (Version 4.10) or META-PPISP (Accessed 2024). After these initial preparations, the proteins were uploaded to docking servers like HADDOCK (Version 2.4) or Cluspro (Version 2.0) to help anticipate protein-protein interactions and docking conformations. The analysis of the docking results shed light on the interaction bonds between SOCS6 and EPOR, revealing important information about their possible binding and functional interactions.

### 2.2. Cell Lines and Cell Culture

We selected two colorectal cancer cell lines: HT-29 to represent moderately differentiated cancer cells and COLO 320DM to represent more aggressive and poorly differentiated cells. The Colon cancer HT-29 cell line was obtained from the American Tissue Culture Collection (ATCC, Manassas, VA, USA). COLO 320DM cell line was a generous gift from Dr. Yahya Tamimi (SQU, College of Medicine and Health Science, Biochemistry Department). HT-29 and COLO 320DM cells were cultured in DMEM and RPMI media, respectively (Gibco, Thermo Fisher Scientific, Waltham, MA, USA) and supplemented with 10% FBS (Gibco, Thermo Fisher Scientific, Waltham, MA, USA) and 1% antibiotic (Gibco, Thermo Fisher Scientific, Waltham, MA, USA).

### 2.3. SOCS6 and EPOR Knockdown Using siRNA

For gene silencing, negative control siRNA (NC) and two SOCS6 siRNAs: s17775 siRNA and s17776 siRNA (Ambion, Austin, TX, USA) were used to downregulate SOCS6 (with different concentrations from 2.5 nM–20 nM) according to the manufacturer’s procedures. Moreover, two EPOR siRNAs: s4765 and 7816 siRNA (Ambion, Austin, TX, USA) were used to silence EPOR (with different concentrations from 5 nM–60 nM). HT-29 (6 × 10^5^) and COLO 320DM (8 × 10^5^) cells were seeded in a six-well plate and transfected the next day using Lipofectamine RNAiMAX (Invitrogen, Thermo Fisher Scientific, Waltham, MA, USA) as a transfection reagent according to the manufacturer’s procedure.

### 2.4. RNA Extraction and Quantitative RT-PCR

Total RNA was extracted from HT-29 and COLO 320DM cells using the Purelink RNA mini kit (Invitrogen, Thermo Fisher Scientific, Waltham, MA, USA) according to the manufacturer’s instruction. Then, cDNA was synthesized using a high-capacity cDNA reverse transcription kit with RNase inhibitors (Applied Biosystems, Thermo Fisher Scientific, Waltham, MA, USA) according to the manufacturer’s protocols and stored at −20 °C before gene quantification. Gene expression was assessed by RT-qPCR using gene-specific primers (Table 1), assays with Power Up SYBR Green master mix (Applied Biosystems, Thermo Fisher Scientific, Waltham, MA, USA) using an Applied Biosystems 7900HT fast detection system (Applied Biosystems, Foster City, CA, USA). GAPDH was used as an endogenous control to normalize expression data. Thermal cycling conditions included 95 °C for 10 min and 40 cycles at 95 °C for 15 s and 60 °C for 1 min according to the RT-qPCR protocol. Each transfection experiment was run in triplicate, each sample in RT-PCR was run in duplicates, and 2^−ΔΔCt^ method was used to get relative expression values.

### 2.5. Viability Assay

Cellular viability is assessed by the Neutral Red Uptake (NRU) assay (Sigma Aldrich, Gillingham, UK), that measures the amount of neutral red dye that live cells take in and bind to their lysosomes. Prior to use, the neutral red reagent was diluted 1:65 according to the manufacturer’s instructions, incubated overnight, and filtered the following day. A suitable density of cells is first seeded into a 6-well tissue culture plate. Then, cells were transfected the following day with scramble and SOCS6 siRNA. Cells were trypsinized and counted after 48 h of transfection. They were then seeded (20,000 cells/well) into three 96-well plates (24, 48, and 72 h) and let to incubate overnight to promote adhesion and growth. The cells were gently rinsed with PBS after the incubation process, and the media was then removed. Subsequently, a filtered neutral red dye solution was added, and the plate was incubated for three hours to allow dye uptake by viable cells. Then, cells were washed out gently with PBS, and the dye was extracted using a freshly made de-stain solution (ethanol/glacial acetic acid/distilled water in %: 50/1/49). Once the colour extract was uniform, after approximately 10 min of shaking on a plate shaker, the NRU was fully extracted from the cells. Later, a microplate reader (Synergy H1, BioTek, Winooski, VT, USA) was used to detect the absorbance of the dye solution that had been extracted at 540 nm. The absorbance values were recorded, and mean values for each group were calculated. In order to find the relative cell viability, these results were normalized to the negative control.

### 2.6. Colony Formation Assay

Colorectal cancer cells were seeded in six-well plates and transfected with either scrambled siRNA (control) or SOCS6 siRNA. The SOCS6 siRNA-treated or NC CRC cells (20,000 cells) were subsequently seeded at a low cell density in six-well plates and propagated for 10–14 days. Media was renewed on a twice-weekly basis. The cells were washed with DPBS, stained with crystal violet to visualize the colonies, and images were acquired after a period of 10–14 days of treatment.

### 2.7. Cell Migration Assay

The cells were plated in 6-well plates to achieve around 50–70% confluence the following day. Subsequently, they were transfected with 10 nM SOCS6 siRNA, using the previously described method. Cells were then seeded at a density of 65,000 in each compartment of a 2-well insert (Ibidi, Gräfelfing, Germany) and incubated for 24 h. The inserts were then removed, and the gap closure was monitored at 0, 24, 48 and 72 h. Images of wound closure were captured. The quantification of wound closure was performed using Image J software (Version 1.53t).

### 2.8. Cell Invasion Assay

To assess the effect of SOCS6 on the invasiveness of CRC cells, QCM Collagen cell invasion assay kit (Sigma Aldrich, St. Louis, MO, USA) was used that employs a 24-well plate with 8 um pores and a colorimetric approach. At a density of 100,000 cells per insert in 250 uL of serum free Media, SOCS6 siRNA transfected cells were added to the interior of the insert. The lower chamber was added into 500 uL of medium plus 10% FBS. After two days of incubation, cells which had invaded the lower chamber were stained and treated with extraction solution. Using a Multiscan spectrophotometer, the absorbance was measured at 560 nm (Thermofisher Scientific, Waltham, MA, USA).

### 2.9. Assessment of Apoptosis Using Active Caspase-3 ELISA

The Human Caspase-3 (active) ELISA Kit (Invitrogen, Thermo Fisher Scientific, Vienna, Austria) was used to evaluate apoptosis in this experimental samples. Quantitative measurement of active caspase-3 levels, a critical effector protein in the apoptotic cascade, was achieved in this test by means of a sandwich enzyme-linked immunosorbent method. The cell lysates were extracted with RIPA Buffer (Thermo Fisher Scientific, Waltham, MA, USA), diluted according to the ELISA protocol and then incubated in wells of a microplate that had been coated specifically for caspase-3 in its active state. A substrate solution was added after caspase-3 bound to the capture antibody, and a secondary antibody coupled with horseradish peroxidase was added subsequently. A spectrophotometer was used to quantify the resulting colour intensity at 450 nm, and the concentration of active caspase-3 was ascertained by constructing a standard curve using recombinant caspase-3. This method enabled the exact measurement of apoptotic activity in the samples being studied.

### 2.10. Statistical Analysis

Statistics and data visualization were performed using GraphPad Prism software (version 8.0.2.263). An appropriate statistical test (*t*-test for normally distributed data, Mann-Whitney U for non-normally distributed data) was used based on the data format, and a *p*-value of 0.05 was used as a cutoff for significance.

## 3. Results

### 3.1. Bioinformatics Analysis Revealed SOCS6 Interaction with EPOR

**Figure 1:**
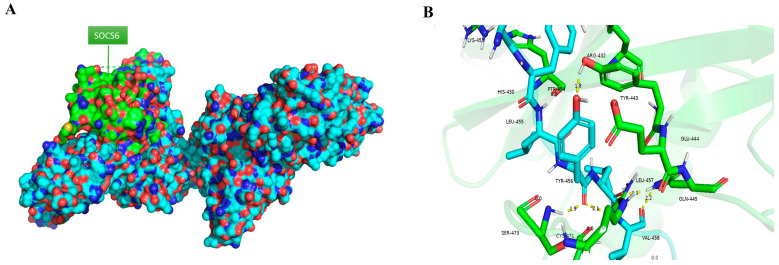
(**A**) Interaction between SOCS6 and EPOR in PyMOL, (**B**) Polar bond between SOCS6 and EPOR (green = SOCS6, blue = EPOR).

Human EPOR sequence that was extracted from UniProt showed three different regions that are previously reported as targets for SOCS6. SOCS6 usually interacts with phosphotyrosine that has tyrosine +1,+2 and +3 valine, isoleucine or tyrosine [34]. The interaction between SOCS6 (PDB:2VIF) and EPOR (PDB:1ern) was illustrated using PyMOL in Figure 1.

### 3.2. Effect of SOCS6 Knockdown on Cellular Proteins and Function

#### 3.2.1. Expression Analysis of SOCS6 and EPOR After SOCS6 Silencing

**Figure 2:**
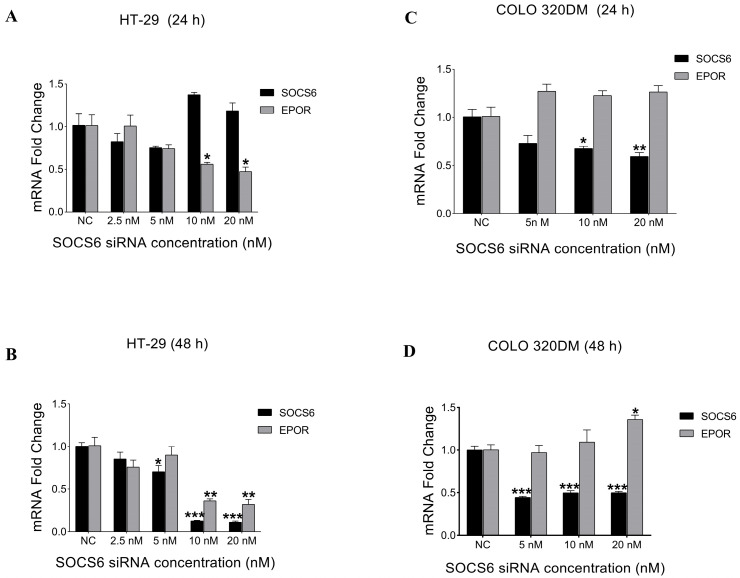
Effect of SOCS6 silencing on the relative expression of SOCS6 and EPOR in colorectal cancer cell lines. Colorectal cancer cells HT-29 (**A**,**B**) and COLO 320DM (**C**,**D**) transfected with different concentrations of SOCS6 siRNA for 24 h (**A**,**C**) and 48 h (**B**,**D**). NC: negative control of cells transfected with scramble siRNA. *p*-values of *t*-test comparing to the NC group: * *p* < 0.05, ** *p* < 0.01, *** *p* < 0.001.

To determine the optimal SOCS6 siRNA concentration, HT-29 and COLO 320DM cells were transfected with different siRNA concentrations (2.5 nM–20 nM) at two different time points (24 h and 48 h). Subsequently, RT-PCR was performed. The results demonstrated that 10 nM was the optimal concentration that showed a significant decrease in SOCS6 expression 48 h post-transfection in both HT-29 and COLO 320DM cells (Figure 2). Furthermore, to determine if SOCS6 regulates EPOR, we quantified EPOR expression. SOCS6 knockdown reduced EPOR expression in HT-29 cells but increased it in COLO 320DM cells, indicating a cell line–dependent regulatory relationship.

#### 3.2.2. SOCS6 Silencing Enhanced Cell Viability in HT-29 and COLO 320DM Cells

**Figure 3:**
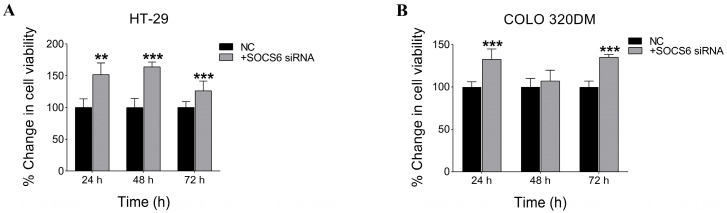
SOCS6 silencing enhanced cell viability in HT-29 and COLO 320DM cells. Effect of SOCS6 silencing on cell viability in colorectal cancer cells. HT-29 (**A**) and COLO 320DM (**B**) cells were transfected with 10 nM SOCS6-targeting siRNA or negative control siRNA, and cell viability was assessed using the Neutral Red Uptake (NRU) assay. A significant increase in cell viability was observed in both cell lines following SOCS6 knockdown (*p* < 0.05). Data represent the mean of three independent experiments. NC: negative control transfected with scramble siRNA. *p*-value of Mann-Whitney test comparing to NC group: ** *p* < 0.01, *** *p* < 0.001.

To assess the effect of silencing SOCS6 on cell viability, an NRU assay was performed. The results showed a significant increase in cell viability following SOCS6 knockdown using 10 nM siRNA in both HT-29 and COLO 320DM cells, implying a tumour suppressive role of SOCS6 (Figure 3).

#### 3.2.3. SOCS6 Silencing Enhanced Colony Formation Ability in HT-29 and COLO 320DM Cells

**Figure 4:**
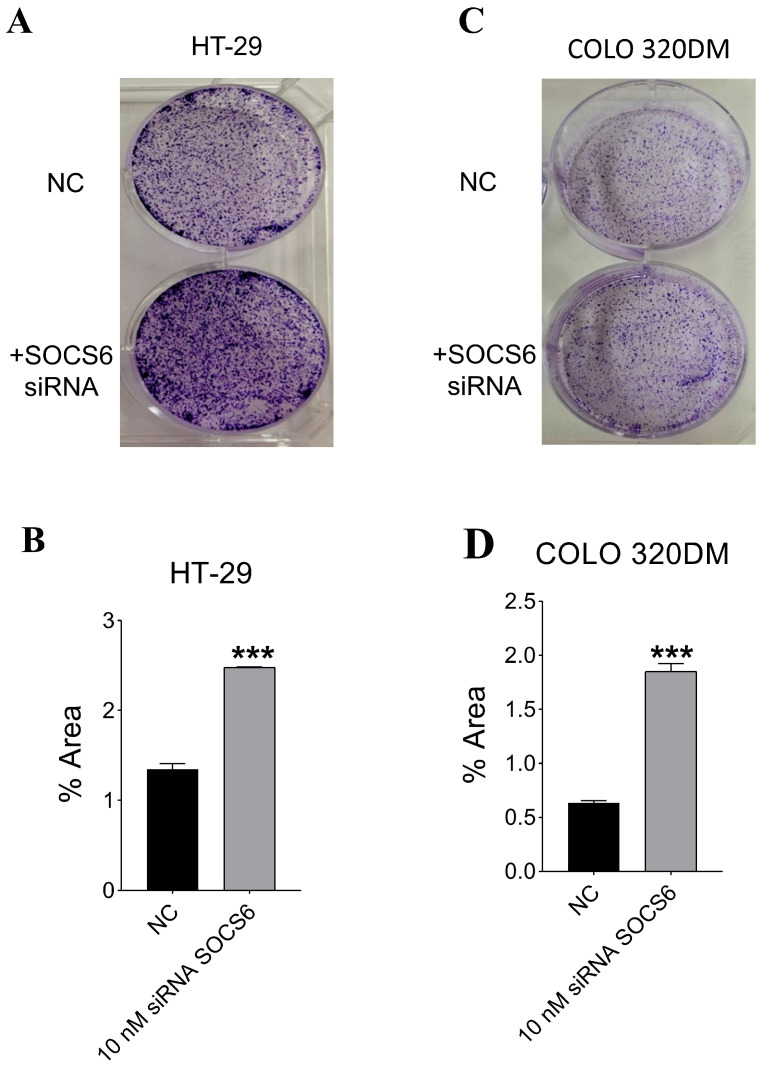
Effect of SOCS6 silencing on clonogenic potential of colorectal cancer cells. HT-29 (**A**,**B**) and COLO 320DM (**C**,**D**) cells were transfected with 10 nM SOCS6-targeting siRNA or negative control siRNA (NC), and colony formation assays were performed to evaluate long-term proliferative capacity. *p*-value of Mann-Whitney (in HT-29) and *t*-test (in COLO 320DM) comparing to the NC group: *** *p* < 0.001.

Colony formation assay was conducted to evaluate the clonogenic survival (combined effects of cell proliferation and cell death) SOCS6-silenced HT-29 and COLO 320DM cells. A marked increase in colony number and size was observed following SOCS6 knockdown compared with the control (Figure 4).

#### 3.2.4. SOCS6 Silencing Enhanced Cell Migration of COLO 320DM Cells and Upregulated Caspase-3 Levels in HT-29 Cells, with Non-Significant Invasive Effects in Both Cells

**Figure 5:**
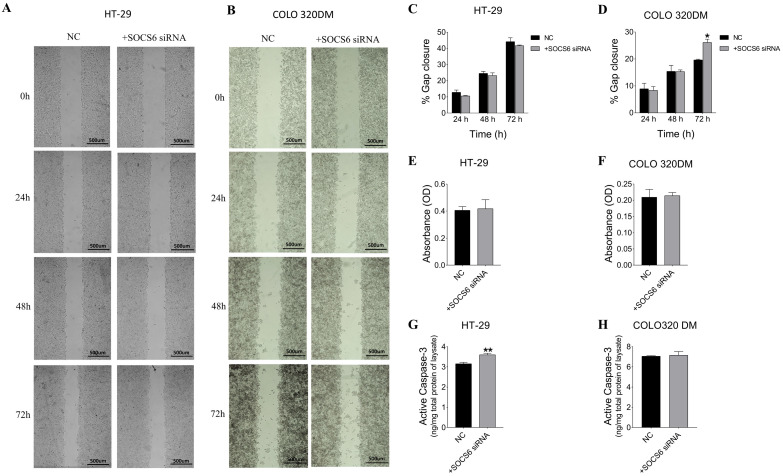
Effect of SOCS6 silencing (10 nM SOCS6 siRNA) on cell migration (**A**–**D**), invasion (**E**,**F**), and active caspase-3 levels (**G**,**H**) in HT-29 and COLO 320DM. Quantitative analysis (**C**,**D**) of the percentage of gap closure is presented for HT-29 and COLO 320DM cells. Invasive capability of HT-29 (**E**) and COLO 320DM (**F**). Quantification of active caspase-3 in HT-29 (**G**) and COLO 320DM (**H**). Data represent the mean of three independent experiments. NC: negative control cells transfected with scramble siRNA. *p* = value of *t*-test applied to compare to the NC group: * *p* < 0.05, ** *p* < 0.01.

To analyse the role of SOCS6 in migration, gap closure was observed in SOCS6-silenced HT-29 and COLO320 DM cells until 72 h (Figure 5). The analysis showed no significant difference in gap closure in HT-29 cells (*p* > 0.05), however significant increase in gap closure was observed in SOCS6-silenced COLO 320 DM cells at 72 h (*p* = 0.031).

To assess the role of SOCS6 on apoptosis, active caspase-3 levels were measured in SOCS6-silenced cells. The results demonstrated a significant increase in active caspase-3 levels in HT-29 cells (*p* = 0.0056), suggesting its involvement in the apoptotic pathway (Figure 5). However, a non-significant increase was observed in COLO 320DM cells post SOCS6 silencing.

The invasive ability of SOCS6-silenced cells was assessed in HT-29 and COLO 320DM cells. The results indicated no significant changes in invasiveness in both cells post SOCS6 siRNA-transfected cells (Figure 5).

### 3.3. Effect of EPOR Knockdown on Cells Viability

#### 3.3.1. Expression Analysis of EPOR and SOCS6 After EPOR Silencing

**Figure 6:**
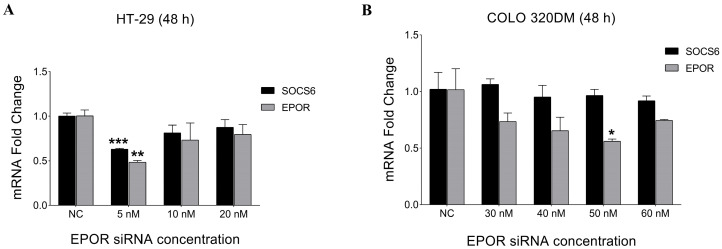
Effect of EPOR silencing on the relative expression of EPOR and SOCS6 in colorectal cancer cell lines. HT-29 cells (**A**) and COLO 320DM (**B**) transfected with different concentrations of EPOR siRNA (48 h). COLO320DM cell lines required ten times more siRNA (50 nM) than HT-29 (5 nM) to significantly knockdown EPOR. NC: negative control group of cells transfected with scramble siRNA. *p*-value of *t*-test was applied: * *p* < 0.05, ** *p* < 0.01, *** *p* < 0.001.

To determine the optimal EPOR siRNA concentration, HT-29 and COLO 320DM cells were transfected with different siRNA concentrations (5 nM–60 nM) for 48 h. Subsequently, RT-PCR was performed. The results demonstrated that 5 nM was the optimal concentration that showed a significant decrease in EPOR expression in HT-29. However, 50 nM EPOR siRNA concentration was the most efficient in silencing COLO 320DM cells (Figure 6). SOCS6 expression was quantified after EPOR knockdown, revealing a significant decrease in HT-29 cells but no change in COLO 320DM cells.

#### 3.3.2. EPOR Silencing Enhanced Cell Viability in HT-29 and COLO 320DM Cells

**Figure 7:**
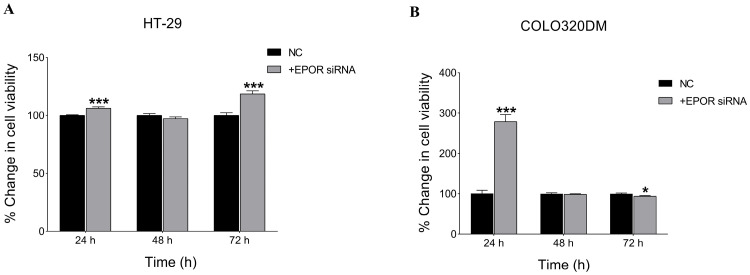
Effect of EPOR silencing on cell viability in colorectal cancer cell lines. Viability of HT-29 (**A**) and COLO 320DM (**B**) cells was assessed following transfection with EPOR-targeting siRNA at indicated concentrations. Cell viability was determined using the Neutral Red Uptake (NRU) assay at 24 h, 48 h and 72 h post-transfection to evaluate the effect of EPOR knockdown on cell viability. NC: negative control group of cells transfected with scramble siRNA. *p*-value of *t*-test was applied: * *p* < 0.05, *** *p* < 0.001.

To assess the effect of silencing EPOR on cell viability, an NRU assay was performed. The results showed a significant increase in cell viability following EPOR knockdown using 5 nM siRNA in HT-29 and 50 nM siRNA in COLO 320DM cells by 24 h post-transfection (Figure 7). However, by 72 h after knockdown, cell viability was higher in HT-29 cells, while COLO 320DM cells displayed reduced viability, suggesting a cell-specific effect of EPOR.

## 4. Discussion

**Figure 8:**
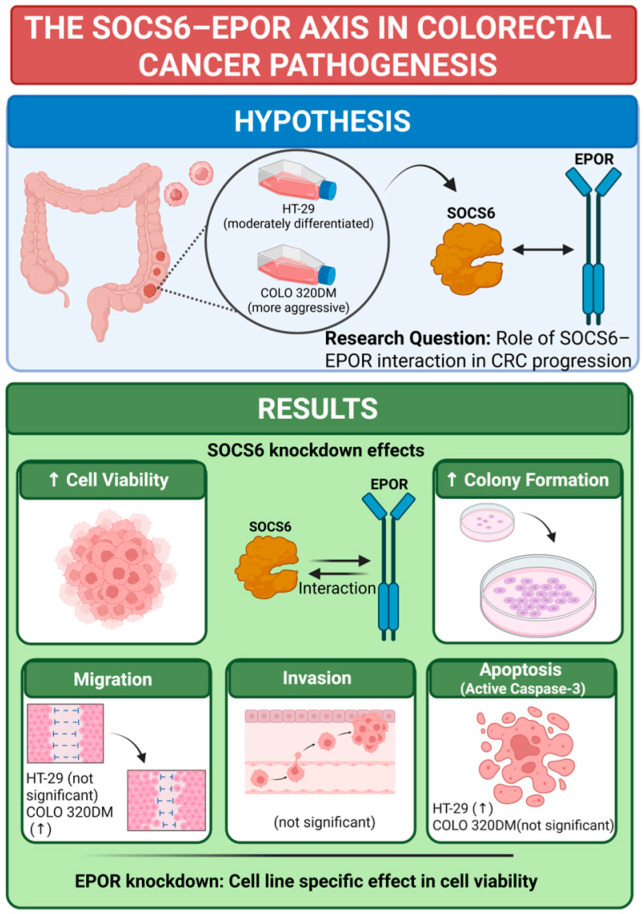
Conclusion of SOCS6 Knockdown effect in colorectal cancer cells. Knockdown experiments were performed, and effect on cell viability, colony formation, migration, invasion and active caspase-3 was investigated. EPOR knockdown was performed as well, and the cell viability was examined in same cell lines (HT-29 and COLO 320DM). ↑ = increase. BioRender was used to create the figure (Created in BioRender. a, A. (2025) https://BioRender.com/rp1tx94 (accessed on 22 December 2025)).

This study aimed to investigate SOCS6 expression and elucidate its association with EPOR in colorectal cancer. The summary of the results is illustrated in Figure 8.

In our previous work using dot blot analyses (not published), phospho-peptides of tyrosine kinase receptors were used to identify bindings to the SOCS6-SH2 domain. These analyses revealed that SOCS6 can bind to multiple candidates, including the erythropoietin receptor (EPOR), a tyrosine kinase receptor. Further studies have shown that SOCS6 exhibits a preference for the amino acids tyrosine, isoleucine, and valine at positions pY+1 to pY+3 relative to the phosphor-tyrosine [34]. These specific residues are also present in EPOR as shown in Figure 1. According to the UniProt database, these sites in EPOR are known to interact with STAT1, STAT3, and STAT5. Therefore, it is predicted that these sites serve as the interaction points between SOCS6 and EPOR.

At 48 h’ post-transfection, 10 nM siRNA significantly reduced SOCS6 expression in HT-29 and COLO 320DM colorectal cancer cells, indicating it as the optimal concentration for SOCS6 silencing. Notable interplay between cell lines is suggested by the fact that HT-29 and COLO 320DM cells regulate EPOR expression differently after SOCS6 knockdown. There may be a positive regulatory link or reliance in HT-29 cells, as EPOR expression was reduced upon SOCS6 knockdown. Due to the absence of functional EPOR signalling in most HT-29 cells, this may indicate transcriptional regulatory mechanisms or indirect stabilization [58]. The observed increase in EPOR expression in COLO 320DM following SOCS6 knockdown suggests an inhibitory effect of SOCS6 on EPOR, which is in line with SOCS6’s known role as a negative regulator of cytokine signalling pathways [3,17]. Similarly, SOCS family proteins are known to play a dual role in regulating receptor expression and the results of downstream signalling through context-dependent feedback mechanisms [2,59,60]. Such opposing effects highlight cell context-dependent regulatory networks [61]. In hepatocellular carcinoma, PETEN, a tumour suppressor gene connected to SOCS6, showed a compensating situation-dependent impact [62]. Many microRNAs target both PTEN and SOCS6, suggesting that these two genes are involved in similar regulatory mechanisms [62] SOCS6 loss may show compensatory receptor upregulation in COLO 320DM to enhance PI3K/Akt survival signalling, whereas in HT-29, the network might suppress receptor levels to maintain cellular homeostasis, and this is beyond the scope of this current study. These findings resonate with reports of SOCS proteins exerting selective regulation in different cancer types [3].

Consistent with SOCS6’s tumour suppressive effect, HT-29 and COLO 320DM cells showed a marked increase in cell viability following SOCS6 knockdown. SOCS6 controls cell proliferation and survival by negatively regulating oncogenic pathways like PI3K/Akt and MAPK/ERK [63]. The silencing of SOCS6 disables this inhibitory regulation, resulting in augmented proliferation and viability, in accordance with prior research indicating that the absence of SOCS6 is associated with heightened cancer cell growth [17]. Parallel findings have been documented, indicating that the inhibition of SOCS family members promotes tumour progression by enhancing proliferative signalling [17,64,65]. These findings are in line with previous research that has shown that inhibiting SOCS proteins in various malignancies increases cell survival and proliferation. This research emphasises the importance of SOCS6 as a negative regulator of tumour progression [66,67].

Colony formation assays assess the proliferative and survival capacity of cancer cells, key indicators of tumourigenicity and cancer progression. The finding that HT-29 and COLO 320DM cells exhibit increased colony formation upon SOCS6 knockdown further supports SOCS6’s tumour suppressive function in controlling the progression of colorectal cancer [68]. In line with SOCS6’s known role as a negative regulator of oncogenic signalling pathways, the increased colony formation upon SOCS6 silencing predicts improved self-renewal and clonogenic potential. Many investigations have shown that SOCS6 suppresses pathways like PI3K/Akt and MAPK/ERK, which are often elevated in colorectal cancer and other cancers to promote cell growth and survival [63,69,70,71,72]. By eliminating this regulatory control, SOCS6 loss enhances colony formation by promoting cell cycle progression and reducing apoptosis through higher phosphorylation of downstream effectors [3,68].

Cell migration assays were performed to evaluate migratory capacity after SOCS6 knockdown where COLO320DM cells demonstrated a significant enhancement in migration, supporting the hypothesis that SOCS6 serves as a crucial inhibitor of migratory potential in more aggressive colorectal cancer subtypes. This is consistent with other studies identifying SOCS6 as an inhibitory regulator of epithelial-mesenchymal transition and cellular migration in colorectal cancer [3,73]. The increased migration noted in COLO 320DM cells is probably facilitated by the activation of downstream signalling pathways, such as ERK/MAPK, which encourage cytoskeletal reorganization and invasive characteristics subsequent to SOCS6 depletion [67,68,74]. Conversely, suppressing SOCS6 did not yield a significant alteration in the migration of HT-29 cells, suggesting that under the examined conditions, SOCS6 may not exert a substantial regulatory influence on motility in this cell line. HT-29 cells possess an intrinsically limited migratory ability and unique signalling patterns that restrict the immediate effects of SOCS protein suppression on migration and gap closure [58]. This epithelial-like phenotype is associated with diminished expression of EMT markers and reduced aggressiveness, potentially elucidating the absence of response to SOCS6 knockdown.

Notably, despite the variations in migration, SOCS6 knockdown did not substantially affect the invasiveness of either HT-29 or COLO320DM cells. This indicates that although SOCS6 regulates proliferation and migration, its direct impact on invasion may be limited or necessitate collaboration with other molecular elements. This aligns with previous research indicating that microRNAs such as miR-301a can promote invasion by downregulating SOCS6; however, the resultant invasive phenotype frequently relies on supplementary cofactors and the cellular environment [74]. The findings are in line with the current thinking that complex networks of signal transduction and micro-environmental factors influence cancer metastasis and invasion, with SOCS6 having a role but not the only one [68,73]. Further investigation of SOCS6’s role in invasion requires combinatorial gene perturbation studies and comprehensive analysis of EMT markers.

SOCS6 silencing did not result in significant changes in active caspase-3 levels or invasive capacity in the colorectal cancer cell line COLO 320DM. Nevertheless, these cells exhibited enhanced clonogenic potential and increased two-dimensional migratory ability. This pattern suggests the presence of a survival-optimized cellular phenotype, likely associated with the poorly-differentiated status of COLO 320DM cells [75]. Significantly elevated active caspase-3 in HT-29 cells but not in COLO 320DM cells, indicating cell line–specific regulation of apoptosis. The absence of a similar effect in COLO 320 DM cells suggests that SOCS6-mediated regulation of apoptosis is cell line–specific, potentially influenced by differences in differentiation status, survival signalling pathways, or compensatory mechanisms that bypass caspase-3 activation. Poorly differentiated cancer cells like COLO 320DM lost many normal epithelial features and often display aberrant signalling pathways like impaired Caspase-3 activation that contributes to resistance to apoptosis, increased survival, and more aggressive behavior. The caspase-3 paradoxically has non-apoptotic capabilities that can enhance proliferation and tumour growth in some conditions, in addition to its crucial role in cancer [76,77]. A non-apoptotic function of caspase-3 in cancer is revealed that oncogene-driven malignant transformation is aided by caspase-3 activation via a pathway involving Endonuclease G (EndoG). This, in turn, promotes tumourigenic signalling through Src kinase-mediated phosphorylation of STAT3 [76]. Beyond its traditional role in apoptosis, caspase-3 contributes to the advancement of cancer, highlighting its dual function. Consistent with research showing caspase-3’s role in colorectal cancer survival and proliferation pathways, the increased caspase-3 in HT-29 cells is probably due to compensatory responses or non-canonical signalling rather than traditional apoptosis activation [77]. The genetic or epigenetic modifications that promote survival in the face of SOCS6 reduction may explain why the caspase-3 response is reduced in COLO 320DM cells [68].

EPOR knockdown is a key approach to investigate erythropoietin signalling in colorectal cancer development and its potential interaction with SOCS6. Finding the optimal dose of EPOR siRNA showed that different conditions were needed to successfully silence genes in HT-29 cells compared to COLO 320DM cells. After 48 h, HT-29 cells showed significant EPOR mRNA knockdown at a dose of 5 nM, but COLO 320DM needed a much higher concentration of 50 nM to accomplish the same type of silence. Variations in transfection efficiency and/or cell line-specific variability in EPOR transcript quantity are likely to blame for this discrepancy. The Human Protein Atlas indicates that HT-29 cells exhibit reduced basal EPOR expression; hence, a lower dosage of siRNA is necessary for effective degradation, consistent with the assumption that lower target mRNA levels require fewer siRNA molecules [78]. On the other hand, in order to achieve sufficient knockdown in COLO 320DM cells with elevated EPOR expression, more siRNA input is required. Moreover, a study found that primary tumour cells from human breast, non-small cell lung, colorectal, and ovarian tissues, including the HT-29 colorectal cell line, express minimal to no functional erythropoietin receptor (EpoR) protein and do not show Epo-induced signalling pathways. Due to the lack of functional EpoR in HT-29 cells, the paper’s findings suggest that knocking down EPOR would not directly cause a reduction in SOCS6; the observed effect is likely an indirect consequence, such as pathway cross-talk or off-target effects of the knockdown reagent [58].

Different cell-specific regulatory networks are further highlighted by the post-knockdown evaluation of SOCS6 expression. Following EPOR silencing, SOCS6 mRNA levels dropped significantly in HT-29 cells, indicating that EPOR-mediated signalling pathways are at least largely responsible for SOCS6 transcription. The roles of SOCS proteins as feedback inhibitors, which are transcriptionally controlled in response to cytokine receptor activation, are consistent with this [65,79]. It appears that there are other regulatory mechanisms that keep SOCS6 levels stable in COLO 320DM cells, as there were no notable changes in SOCS6 expression following EPOR knockdown. These mechanisms could include compensatory signalling pathways or constitutive expression that is not dependent on EPOR. It was reported that alternative transcription factors like AP-1 and SP1/SP3 can drive SOCS gene expression, maintaining levels if EPOR-driven STAT activation isn’t present [2]. Cells also express other receptor tyrosine kinases (RTKs), which can influence each other’s activity, leading to sustained regulation of downstream targets like SOCS6 if EPOR signalling is blocked [65,80].

Optimizing knockdown efficacy while minimizing off-target effects requires adjusting siRNA dosage to intrinsic cellular context and gene expression levels [81]. These findings emphasise the significance of this approach. It is worth conducting additional mechanistic research to understand the relationships between EPOR and SOCS6 in different forms of colorectal cancer. The fact that SOCS6 was modulated in HT-29 cells but not COLO 320DM cells suggests that there are cell-type-specific changes in the regulation of feedback and cytokine signal communication.

There was a complicated, line-specific, biphasic response to EPOR knockdown in colorectal cancer cells during our inquiry into the role of EPOR in cell viability; this contradicts the traditional understanding of EPOR as a straightforward pro-survival protein. A notable increase in cell viability was observed in both HT-29 and COLO 320DM cells 24 h after transfection, which goes against the assumptions derived from studies in other cancer models where EPOR silencing affects viability, such as breast cancer. This unexpected early effect can be explained by a brief compensating mechanism that maintains cellular homeostasis by temporarily upregulating alternative pro-survival pathways like PI3K/Akt or STAT5. Another known concern of siRNA research is off-target effects, which could explain the observed result at the dosages utilized [81]. Consistent with previous research demonstrating that EPOR’s function varies between cell types and environments, the 72-h cell viability divergence further highlights the context-dependent character of EPOR signalling [58]. The fact that the increased vitality remained in HT-29 cells indicates that the initial compensatory signalling was stronger or more long-lasting in this particular line. When compared to the predicted pro-survival function of EPOR, the 72-h decrease in COLO 320DM cell viability is more in line with expectations. Because the initial compensating mechanisms have probably been exhausted, the dependence of COLO 320DM cells on EPOR signalling for prolonged viability is revealed by this delayed effect. The various reactions shown in HT-29 and COLO 320DM cells are in line with what is known as cancer heterogeneity, which is when tumours of the same histological type can display varying molecular characteristics and behaviours. The molecular variations between these two cell lines that would explain their different long-term consequences following EPOR knockdown and the precise signalling pathways involved in this biphasic response at various time points will be the subject of future research.

Despite the fact that this work gives new light on the SOCS6-EPOR axis’s function in colorectal cancer, it is important to note that it has a number of limitations. Experiments were conducted in two-dimensional (2D) model, which might not accurately represent the complexity of the tumour heterogeneity, micro-environment, and immunological interactions that exist in a living organism. To confirm these results, there is ongoing work in human clinical samples to elucidate the SOCS6/EPOR interaction in colorectal cancer. Moreover, in vivo animal models could be a good choice for further confirmation. For better understanding the molecular interaction of SOCS6/EPOR signalling and to evaluate its therapeutic potential in colorectal cancer, future research should incorporate in vivo models, patient samples and proteomic analysis. Further, moving from traditional (2D) culture into more physiologically relevant three-dimensional (3D) models like organoids could enhance the clinical relevance of future studies by offering more precise pictures of the tumour architecture and pharmacological responses. Moreover, we acknowledge that using two colorectal cancer cell lines with distinct differentiation states may contribute to cell line–specific regulatory inconsistencies. Differences in differentiation, genetic background, and signalling network activation can lead to variable responses to SOCS6 or EPOR modulation. HT-29 cells, being more epithelial and adherent, may respond differently in proliferation, apoptosis, or signalling assays compared with the more aggressive, COLO 320 DM cells. Nevertheless, including both cell lines provides a broader perspective on the functional effects of SOCS6 and EPOR across colorectal cancer phenotypes, highlighting context-dependent regulatory mechanisms that may reflect tumour heterogeneity in vivo.

This study found that the SOCS6-EPOR axis could be a target for colorectal cancer (CRC) treatments. Given SOCS6’s tumour-suppressing function, attempts to restore or enhance SOCS6 expression or activity may slow the growth of CRC, especially in the tumours where SOCS6 level are low. At the same time, patient stratification is crucial when thinking about SOCS6-regulated pathways. Recent developments in targeted delivery systems, including platforms based on nanoparticles or siRNA, have opened up new possibilities for the precise manipulation of SOCS6 or EPOR in colorectal cancer. These findings provide support for the translational potential of colorectal cancer therapy programs centered around SOCS6, but additional in vivo and clinical validation is still needed.

## 5. Conclusions

SOCS6 and EPOR exhibit a cell line–specific, bidirectional regulatory relationship in colorectal cancer. SOCS6 knockdown increased EPOR in COLO 320DM but decreased it in HT-29 cells, while EPOR silencing affected SOCS6 only in HT-29 cells, indicating context-dependent feedback. These findings highlight the impact of tumour heterogeneity on cytokine receptor regulation and underscore the need to investigate the underlying molecular pathways to understand their roles and therapeutic potential.

## Figures and Tables

**Table 1:** Primers used in RT-qPCR.

Primer	Sequence
SOCS6 primers (forward)	5′ ATCACGGAGCTATTGTCTGGA
SOCS6 primers (reverse)	5′ CTGACTCTCATCCTCGGGGA
GAPDH primer (forward)	5′ GGATTTGGTCGTATTGGG
GAPDH primer (reverse)	5′ GGAAGATGGTGATGGGATT
EPOR primer (forward)	5′ CAAGTTCGAGAGCAAAGCGG
EPOR primer (reverse)	5′ TTCCTCCCAGAAACACAAG

## Data Availability

The original contributions presented in this study are included in the article.

## Abbreviations

The following abbreviations are used in this manuscript:

ATCC	American Tissue Culture Collection
CEA	Carcinoembryonic antigen
CRC	Colorectal cancer
EndoG	Endonuclease G
EPOR	Erythropoietin receptor
ESAs	Erythropoiesis-stimulating agents
MSI	Microsatellite instability
NC	Negative Control
NRU	Neutral Red Uptake
SOCS	Suppressor of cytokine signalling
SOCS6	Suppressor of cytokine signalling 6
2D	Two-dimensional
3D	Three-dimensional
WHO	World Health Organization
